# Amino Oxidase Hard Protein Corona with Metabolic-Triggered Intracellular Biocatalysis

**DOI:** 10.3390/ijms27167492

**Published:** 2026-08-21

**Authors:** Federica Tonolo, Mary Bortoluzzi, Graziano Rilievo, Alessandro Cecconello, Aura Cencini, Lavinia Rutigliano, Maria Pia Rigobello, Maria Luisa Di Paolo, Alberto Macone, Pasquale Fino, Enzo Agostinelli, Massimiliano Magro, Fabio Vianello

**Affiliations:** 1 Department of Comparative Biomedicine and Food Science, University of Padua, Viale dell’Università 16, 35020 Legnaro, Italy; mary.bortoluzzi@phd.unipd.it (M.B.); graziano.rilievo@unipd.it (G.R.); aura.cencini@phd.unipd.it (A.C.); fabio.vianello@unipd.it (F.V.); 2 Department of Molecular and Translational Medicine, University of Brescia, Viale Europa 11, 25123 Brescia, Italy; alessandro.cecconello@unibs.it; 3 Department of Molecular Medicine, Sapienza University of Rome, Viale Regina Elena 291, 00161 Rome, Italy; laviniarutigliano91@gmail.com; 4 Department of Biomedical Sciences, University of Padua, Via Ugo Bassi 58/b, 35121 Padova, Italy; mariapia.rigobello@unipd.it; 5 Department of Molecular Medicine, University of Padua, Via Gabelli 63, 35121 Padova, Italy; marialuisa.dipaolo@unipd.it; 6 Department of Biochemistry Science “A. Rossi Fanelli”, La Sapienza University of Rome, Viale Regina Elena 332, 00161 Rome, Italy; alberto.macone@uniroma1.it; 7 Department of Sensory Organs, La Sapienza, University of Rome, Policlinico Umberto I, Viale del Policlinico 155, 00161 Rome, Italy; pasquale.fino@gmail.com (P.F.); enzo.agostinelli@uniroma1.it (E.A.); 8 International Polyamines Foundation ‘ETS-ONLUS’, Via del Forte Tiburtino 98, 00159 Rome, Italy

**Keywords:** enzyme delivery, oxidative stress, enzyme therapy

## Abstract

A hard protein corona was engineered onto tannic acid-modified magnetic nanoparticles (SAMN@TA), a magnetic and luminescent core–shell nano-carrier, using bovine serum amine oxidase (BSAO), an enzyme catalyzing the oxidation of polyamines and producing the corresponding aldehydes and hydrogen peroxide. The absorption and intracellular bioactivity of the self-assembled multimodal SAMN@TA@BSAO were investigated on an intestinal barrier model built with human colorectal adenocarcinoma (Caco-2) cells. The tailored BSAO corona possessed fouling resistance and, at the same time, was able to activate the clathrin-mediated endocytosis (CME) mechanism. Despite its size and intrinsic complexity, the nano-vehicle was effectively transported across the cell layer, safely transiting across the cell cytoplasm and reaching the lumen. As a function of intracellular polyamine concentration, the system’s biological activity induced intracellular oxidative stress, leading to the activation of the Keap1/Nrf2 oxidative protection pathway. The SAMN@TA@BSAO effect was well described by a dose–response curve with an EC_50_ of around 30 µg mL^−1^ and a programmable killing efficiency (>50.0%), recalling the feasibility of a low molecular weight drug administration. The present study contributes to the nascent knowledge on engineering protein corona as a key to rationally design nanomaterials for biomedical applications.

## 1. Introduction

Nanotechnological devices were proposed for the development of novel biomedical tools for a wide range of diseases, including viral infections, cardiovascular, and neoplastic diseases [1,2,3,4,5,6,7,8]. The fight against cancer, in spite of significant progress in medical research, is still one of the most challenging human health issues, enticing a constant demand for innovative treatment strategies [9,10,11]. Among these, an attractive alternative to conventional anticancer drugs is represented by enzyme-based therapies targeted to metabolic specificities of cancer cells [12]. In this context, altered metabolic profiles can be used to selectively kill specific cancerous cells, hence minimizing damage to healthy tissues [13].

In some tumors, increased proliferation is associated with polyamine overproduction, making amine oxidases (AOs) elective candidates as therapeutic enzymes for the selective elimination of cancerous cells [14]. The AO family comprises a large class of polyamine-oxidizing enzymes, which are widespread in nature. AOs are classifiable on their specific catalytic process, structural features, locations within the cell, specificity for substrates, and sensitivity to inhibitors [15]. The AO substrates, polyamines, are ubiquitous polycationic alkylamines involved in numerous biological processes, including cell growth and division, cell differentiation, gene expression control, and cell movement [16]. Cellular polyamine levels are extremely variable and are regulated by specific enzymes that catalyze their synthesis and degradation. Spermine (SPM), spermidine (SPD), and putrescine (PUT) are the most abundant polyamines in animal tissues and, depending on the specific cell type, they are present at micro- to millimolar concentrations [17]. High intracellular polyamine levels are produced by dysregulated metabolism and enhanced uptake from the extracellular environment, such as MYC-driven upregulation of biosynthetic enzymes such as ornithine decarboxylase, increased arginase activity in cancer-associated fibroblasts, and glutamine utilization for polyamine synthesis in nutrient-depleted environments [18,19].

As a potential therapeutic enzyme, bovine serum amine oxidase (BSAO) was proposed for strategically inducing cytotoxicity in human tumor cells, including colon adenocarcinomas and melanomas, known for their extreme resistance to treatments [20].

BSAO is a 180 kDa homodimer glycoprotein containing a 2,4,5-trihydroxyphenylalanine quinone (TPQ) organic cofactor and a three histidine-coordinated copper ion, preferentially catalyzing the oxidative deamination of spermine and producing the corresponding aldehyde, in addition to ammonia and hydrogen peroxide (H_2_O_2_) [21,22,23]. Aldehydes and hydrogen peroxide are highly reactive chemical species and are associated with increased oxidative stress in cells [24]. Thus, cells accumulating high concentrations of polyamines were studied for oxidative stress-induced AO-enzyme therapy [24].

Despite the mentioned advantages, the biomedical application of therapeutic enzymes, such as the bulky BSAO protein, is hampered by a lack of cell membrane permeability and a short half-life [12].

In this view, research on nanomaterials focuses on innovative delivery platforms for biomolecules, therapeutic agents, and enzymes, enhancing intracellular transport [25,26,27,28]. In particular, superparamagnetic iron oxide nanoparticles (SPIONs) were found to possess high biocompatibility and interesting magnetic properties [29]. Among SPIONs, surface-active maghemite nanoparticles (SAMNs) exhibit other advantageous features, such as high colloidal stability, easy wet functionalization, and intrinsic fluorescence [30]. Herein, SAMNs were modified with tannic acid (TA) to create a high-affinity nano-carrier for BSAO immobilization and transportation. In this view, it is important to recall that recent studies have demonstrated that the nano-immobilization of an enzyme can improve its stability, substrate accessibility, and even enhance the activity, specificity, and selectivity of the biocatalyst when compared to the soluble enzyme [31]. Stability is conferred by multiple-point binding at the bio–nano interface, resulting in a superior structural stability of the protein. Thus, a very robust hard protein corona was created through a simple self-assembly reaction, preventing the enzymatic cargo from denaturation as well as from the interference of endogenous proteins when tested in actual biological recipients, as substantiated by the registered long-lived biological activity. Furthermore, BSAO corona endowed the nano-vehicle with the ability to cross biological barriers by activating the clathrin-mediated endocytosis pathway, and cytotoxicity was modulated by incubation time, SAMN@TA@BSAO concentration, and, most importantly, polyamine levels, changing from being harmless to detrimental in a predictable way. This stimuli-responsive shell fuels further interest in the application opportunities offered by the rational design of engineered protein coronas.

## 2. Results

### 2.1. Preparation and Characterization of the SAMN@TA@BSAO Nano-Vehicle

**Figure 1:**
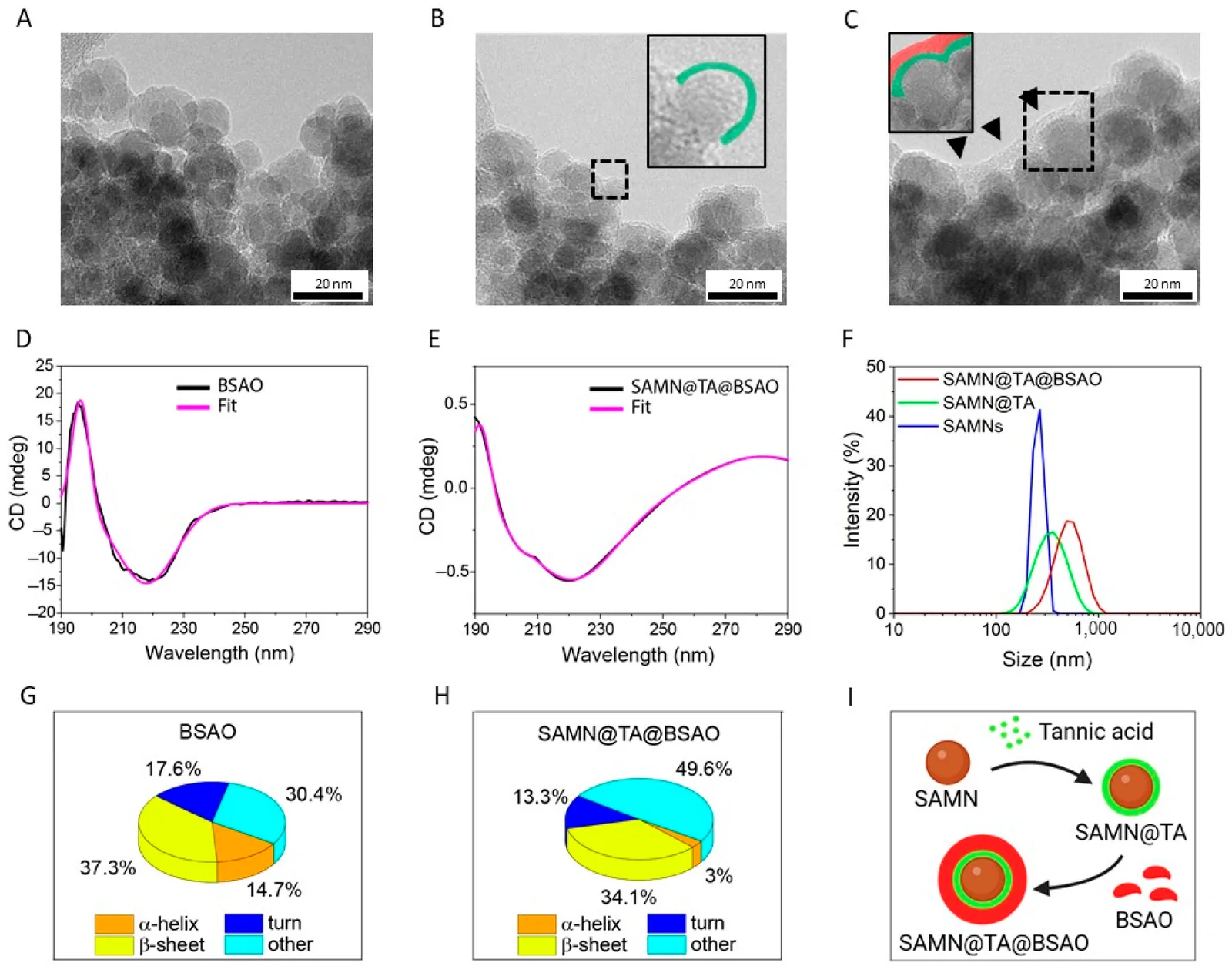
Morphological characterization of the two-step self-assembly reaction and circular dichroism profiles of soluble and nano-immobilized BSAO. (**A**) TEM micrograph of SAMNs. (**B**) TEM micrograph of SAMN@TA; inset: magnification of the TA shell (green). (**C**) TEM micrograph of SAMN@TA@BSAO; inset: magnification of the TA-BSAO double layer shell (green and red, respectively). (**D**) CD profile of native BSAO at pH 7. (**E**) CD profile of SAMN@TA@BSAO. (**F**) Comparison of the hydrodynamic sizes of SAMNs, SAMN@TA, and SAMN@TA@BSAO. (**G**) Secondary structure composition of native BSAO. (**H**) Secondary structure composition of SAMN@TA@BSAO; (orange: α-helix, yellow: β-sheet, blue: turn, light blue: other secondary structures). (**I**) Schematic representation of the stepwise self-assembly of the SAMN@TA@BSAO hybrid. Created in BioRender. BORTOLUZZI, M. (2026) https://BioRender.com/95wfrkx.

SAMN@TA@BSAO ternary hybrid nano-vehicle was obtained by a sequential two-step self-assembly process, using surface-active maghemite nanoparticles (SAMNs) as core material [23]. The final nanohybrid comprised an inner shell of tannic acid (TA) and an outer hard corona exposing bovine serum amine oxidase (BSAO). Figure 1A–C show representative transmission electron microscopy (TEM) micrographs reporting the morphological analysis of the step-by-step self-assembly synthetic sequence (as reported in Figure 1I). Experimental results revealed an increasingly bulkier organic phase associated with the TA inner layer deposition on the parent SAMNs (Figure 1B) and the BSAO outer layer in the ternary nanohybrid (Figure 1C). The first step of the sequence is a dramatic modification of the nanoparticle surface, changing from being stoichiometric crystalline γ-Fe_2_O_3_ to a thick and stable layer of ferric tannates.

This transformation, involving 20% maghemite nanoparticle overall mass, was substantiated elsewhere by Mössbauer spectroscopy and superconducting quantum interference device (SQUID) [32]. The second step represents a mild, wet self-assembly reaction, taking advantage of the well-known tannic acid–protein affinity and the suitable chemical-physical complementarity between BSAO and SAMN@TA.

The successful binding of the enzymatic cargo on SAMN@TA was confirmed by circular dichroism (CD). Figure 1D,E shows the CD profiles of native BSAO and SAMN@TA@BSAO, characterized in both cases by a positive peak at 190–200 nm and a broad negative peak at 210–230 nm. Furthermore, signal deconvolution (Figure 1G,H) showed no substantial sign of denaturation upon nano-immobilization, suggesting tertiary structure preservation of BSAO on SAMN@TA. In this view, the catalytic activity of the nano-immobilized enzyme was checked and found to be well conserved, in good agreement with the CD observations. Anyway, the registered decrease in the α-helix fraction is in agreement with the literature, ascribing the structural feature loss to the chemical interaction at the protein-TA interface [33].

The stepwise size increase during SAMN@TA@BSAO self-assembly was further characterized by dynamic light scattering (DLS) analysis (Figure 1F). The measured hydrodynamic radii of SAMNs, SAMN@TA, and SAMN@TA@BSAO resulted in 265 ± 30 nm, 450 ± 30 nm, and 750 ± 40 nm (Figure 1F, blue, green, and red bars, respectively), compatible with the protein corona thickness of a large macromolecule, such as BSAO. Moreover, the zeta potential (ζ) values of SAMNs, SAMN@TA, and SAMN@TA@BSAO were +30.1 ± 1.4 mV, −28.3 ± 1.7 mV, and −16.1 ± 1.1 mV (conductivity = 0.002 mS/cm, 0.019 mS/cm, and 0.079 mS/cm in water at 22 °C, respectively) (Figure S1). The remarkably high ζ of the parent maghemite nanoparticles reflects the presence of coordinative water saturating the dangling bonds generated by the crystal truncation at the nanomaterial surface. The shift to significant negative ζ values for SAMN@TA upon interaction with TA is compatible with reported tannin coatings at pH 7 [34] and, therefore, with the aforementioned drastic surface reorganization into ferric tannates. From a chemical standpoint, besides the main contribution of hydrogen bonds [33], the binding affinity of BSAO and TA can also be related to the electrostatic interaction between negatively charged TA groups and ionized amino acid side chains, plausibly explaining the decrease, in terms of absolute value, from −28.3 ± 1.7 mV to −16.1 ± 1.1 mV. These results confirm the successful functionalization of the nanoparticles and demonstrate the stability of the colloidal suspension [35]. Indeed, the colloidal suspension of SAMN@TA@BSAO showed no sign of precipitation upon prolonged storage at 4 °C. This is of crucial importance as aggregation can physically hamper the enzyme-substrate interaction by reducing the actual reactive surface exposed to the solvent and, in the worst scenario, can lead to protein denaturation [36]. Moreover, in order to further favor persistence in the medium and, therefore, in the intracellular environment, a nanomaterial concentration in the 5–100 µg mL^−1^ interval was chosen for the following in vitro studies, as previously reported for different biological contexts [37].

### 2.2. Permeability Study of SAMN@TA@BSAO Nano-Vehicle Using a Caco-2 Cells Monolayer

**Figure 2:**
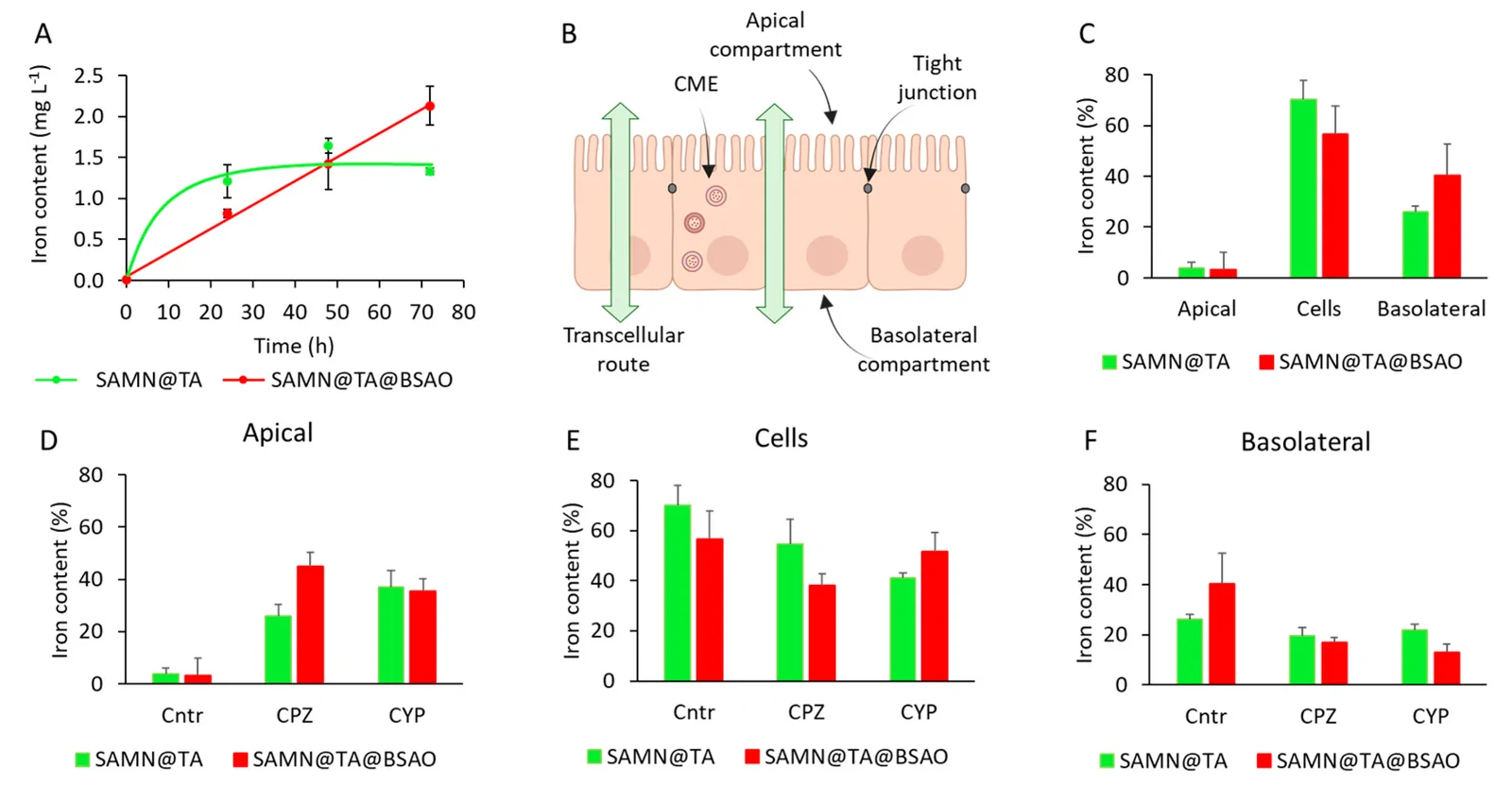
Evaluation of nanohybrid absorption by Caco-2 cells. (**A**) Iron uptake as a function of time using ICP-OES in Caco-2 cell lysates treated with 35 µg mL^−1^ of nanohybrid. Means of at least 3 experiments were compared. (**B**) Schematic representation of the physiological intestinal absorption routes. Created in BioRender. Tonolo, F. (2026) https://BioRender.com/7qeq25l. (**C**) Simulation of intestinal absorption of 35 µg mL^−1^ SAMN@TA or SAMN@TA@BSAO. Cells were treated with SAMN@TA or SAMN@TA@BSAO for 24 h, and then apical, cellular, and basolateral compartments were harvested and subjected to ICP-OES analysis. Means of at least 3 experiments were compared. (**D**–**F**) Iron content evaluation in apical (**D**), cells (**E**), and basolateral (**F**) compartments after simulation of intestinal absorption of SAMN@TA or SAMN@TA@BSAO using 10 µg mL^−1^ chlorpromazine (CPZ) or 5 µg mL^−1^ cytochalasin D (CYP) as inhibitors of clathrin-mediated endocytosis and micropinocytosis/phagocytosis, respectively. The iron content (%) was calculated as the ratio of iron concentration, determined by ICP-OES in every single compartment, to the overall nanoparticle concentration administered to the intestinal model.

Caco-2 cells, derived from human colon carcinoma, are commonly used to assess the permeability of compounds under in vitro conditions, and specifically to simulate absorption across the intestinal barrier. In this view, the protein corona provides the biological identity to a nanomaterial, playing a fundamental role in the nanomaterial trafficking in biological systems [38]. Thus, in order to detail the influence of the tailored BSAO corona on the nano-vehicle fate, inductively coupled plasma optical-emission spectrometry (ICP-OES) was used for determining the uptake rate of the iron-based nano-vehicle core into Caco-2 cells. ICP-OES is a powerful tool for the determination of metals in a variety of different matrices, including biological samples, and was actually already employed to monitor iron oxide nanoparticle internalization in cells [39]. In general, when compared to untreated cells, iron concentration was two orders of magnitude higher upon treatment with nanoparticles. As a control, SAMN@TA internalization showed a saturation behavior, characterized by a fast initial uptake and reaching a plateau upon a 24 h incubation (Figure 2A, green line). Differently, SAMN@TA@BSAO internalization resulted in a time-dependent linear trend (Figure 2A, red line), with an hourly uptake rate of around 1.2 × 10^7^ nanoparticles per cell. Hard corona by engineered BSAO can be meant as a non-self-biological identity, which was reported to be associated with a stealthy character and a different cell uptake behavior [39]. To mimic the intestinal barrier, Caco-2 cells were seeded and treated with 35 µg mL^−1^ SAMN@TA@BSAO or SAMN@TA for 24 h. A model of physiological intestinal absorption was produced, schematically depicted in Figure 2B. Then, the apical, intracellular, and basolateral compartments were harvested and subjected to ICP-OES analysis. Notably, SAMN@TA was prevalently retained in the intracellular compartment, whereas SAMN@TA@BSAO displayed a better ability to cross the biological barrier, as mirrored by its higher concentration in the basolateral compartment (Figure 2C). To further detail the mechanism of nano-vehicle transport across Caco-2 cells, SAMN@TA@BSAO internalization was carried out using chlorpromazine (CPZ) and cytochalasin D (CYP), as inhibitors of clathrin-mediated endocytosis and micropinocytosis/phagocytosis, respectively, being widely used in the investigation of cell uptake mechanisms [38,40,41]. Notably, as reported in Figure 2D–F, SAMN@TA@BSAO transport into the cells was significantly inhibited by CPZ and, to a lesser extent, by CYP, suggesting that the cell uptake of the nano-immobilized enzyme was mainly due to clathrin-mediated endocytosis *via* the transcellular transport route. Indeed, the inhibitor significantly hampered the nanomaterial trafficking, leading to its accumulation in the apical instead of the basolateral compartment, thus inverting the trend observed in Figure 2C. This corroborates the occurrence of a different internalization mechanism between SAMN@TA@BSAO and SAMN@TA.

The intrinsic green fluorescence (Ex. 488 nm, Em. 525 nm) of SAMNs was exploited for determining SAMN@TA@BSAO localization within the cell by confocal microscopy. This unique property allowed monitoring the intracellular localization and uptake of the hybrid nanoparticles without introducing organic fluorophores, which could potentially alter the biological behavior of the nanohybrid. In this view, SAMNs have already been successfully used as vehicles and to monitor the delivery into cells of several biomolecules [30,42,43].

**Figure 3:**
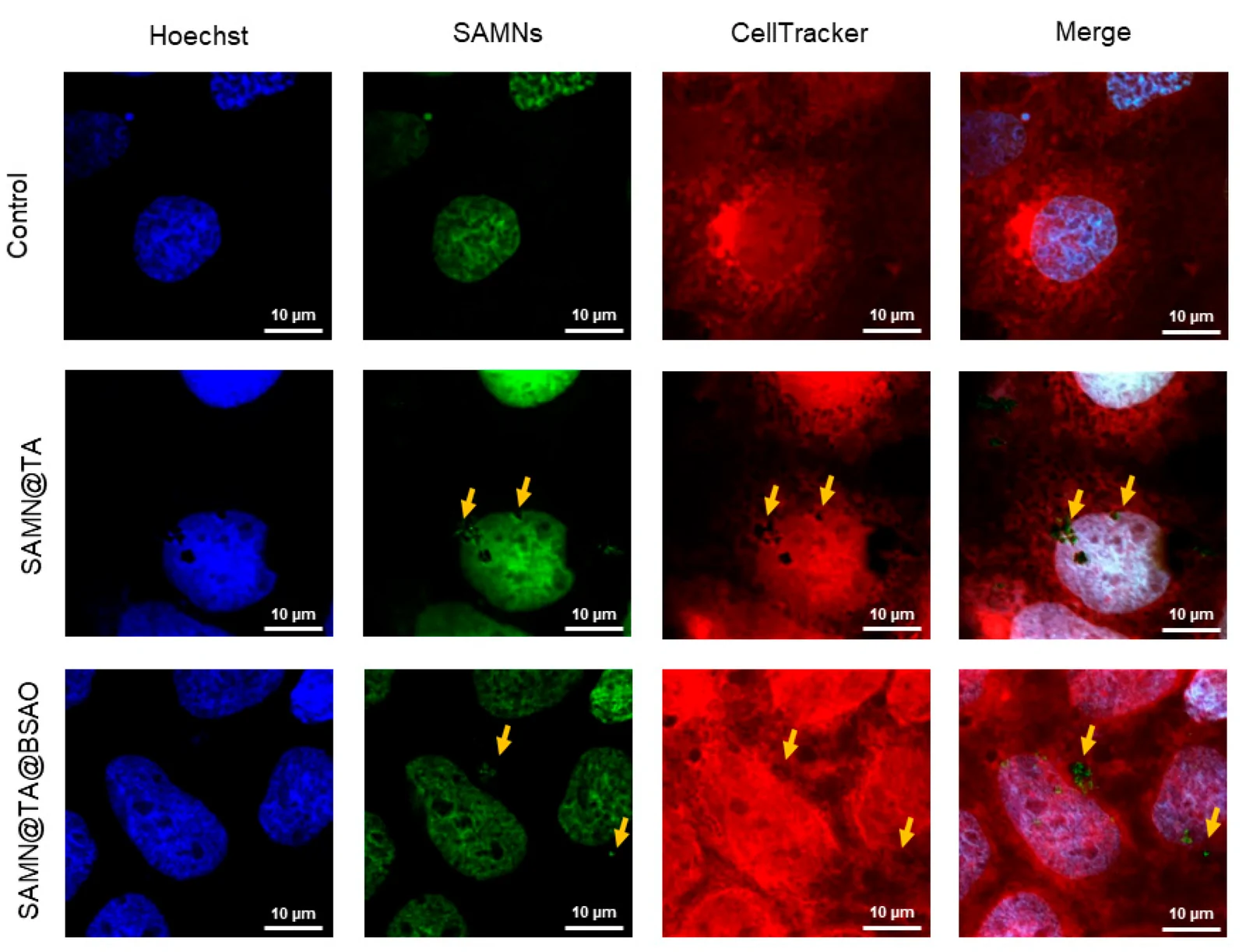
Cellular uptake and localization of SAMN@TA and SAMN@TA@BSAO. Representative confocal stack images (n = 13) of Caco-2 cells treated with 35 μg mL^−1^ SAMN@TA and SAMN@TA@BSAO for 24 h. Cells were stained with Hoechst and CellTracker to highlight the nuclei (blue, λ = 350 nm) and the cytoplasm (red, λ = 555 nm), respectively. In green, SAMN@TA and SAMN@TA@BSAO intrinsic fluorescence after excitation at λ = 488 nm.

As reported in Figure 3, SAMN@TA@BSAO generated green fluorescence can be detected in cells, where the mechanism of uptake appears mediated by vesicle formation. Indeed, as indicated by the yellow arrows, some cytoplasmic and nuclear regions showed dye exclusion in correspondence with the nano-vehicle, as evidenced by CellTracker (red) and Hoechst (blue) staining, respectively, suggesting the presence of vesicles.

### 2.3. Evaluation of SAMN@TA@BSAO Cytotoxicity in Caco-2 Cells

**Figure 4:**
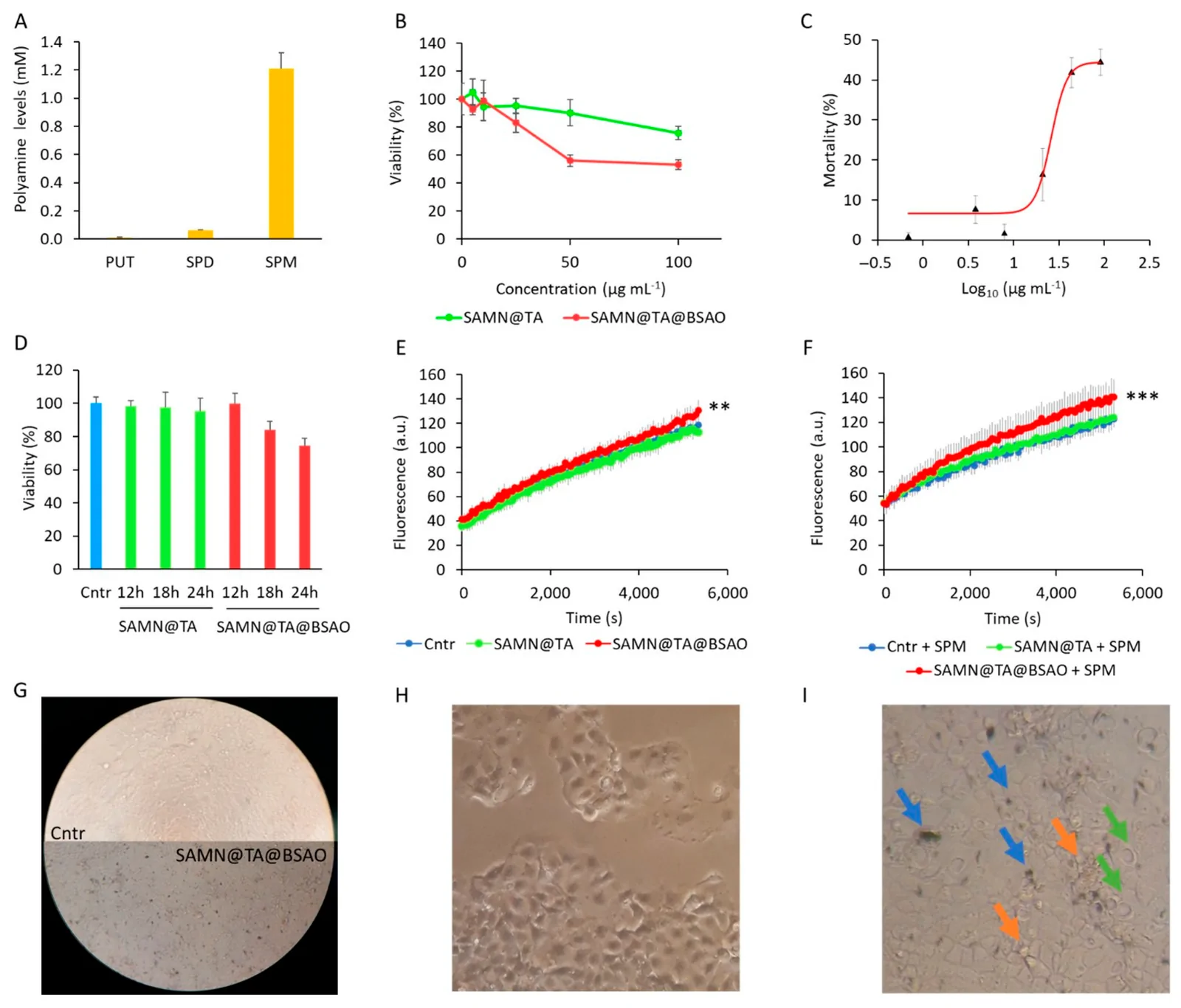
Polyamine abundance and effects of SAMN@TA and SAMN@TA@BSAO on Caco-2 cells. (**A**) Intracellular concentrations of putrescine (PUT), spermidine (SPD), and spermine (SPM) were quantified in Caco-2 cells by tandem gas chromatography–mass spectrometry (GC–MS). PUT: putrescine; SPD: spermidine; SPM: spermine. Means of at least 3 experiments were compared. (**B**) Cell viability was evaluated by MTT assay in Caco-2 cells treated with increasing concentrations of SAMN@TA or SAMN@TA@BSAO for 24 h. (**C**) SAMN@TA@BSAO dose–response curve. (**D**) Cells treated with 35 µg mL - SAMN@TA or SAMN@TA@BSAO at different time points were subjected to the MTT test. Data points are expressed as a percentage of the values observed in the untreated group. Means of at least 3 experiments were compared (8 replicates for each experiment). (**E**,**F**) Evaluation of ROS production in Caco-2 cells treated with 35 µg mL^−1^ SAMN@TA or SAMN@TA@BSAO for 24 h. The reaction was estimated by following dihydro-rhodamine 123 probe fluorescence (Exc. 485 nm; Em. 527 nm) for 1.5 h. The addition of SPM (10 mM) was used to boost the activity of SAMN@TA@BSAO. The reported values are the means of at least 3 experiments (8 replicates for each experiment). (*** *p* < 0.001, ** *p* < 0.01). (**G**) Images of Caco-2 cells untreated (upper figure) and treated (bottom figure) with SAMN@TA@BSAO 35 µg mL^−1^ for a total of 24 h, respectively, were acquired with a ZEISS TELAVAL 31 microscope (ZEISS Italia, Reggio Emilia, Italy) with a 10× objective. (**H**) Higher magnification image of untreated Caco-2 cells acquired with a 40× objective. (**I**) Higher magnification image of treated Caco-2 cells acquired with a 40× objective. Blue arrows indicate SAMN@TA@BSAO hybrid, orange arrows highlight marked cell nuclei, and green arrows indicate vacuoles.

Since the catalytic activity of BSAO promotes the oxidation of endogenous polyamines into toxic aldehydes and hydrogen peroxide, the viability of SAMN@TA@BSAO-treated Caco-2 cells was monitored to gather insights into the nano-vehicle intracellular activity. As shown in Figure 4A, the most abundant polyamines in Caco-2 cells were spermine (SPM), 1.2 ± 0.1 mM, spermidine (SPD), 59 ± 6 µM, and putrescine (PUT), 11 ± 1 µM. Notably, spermine is the ideal BSAO molecular target [20]. While these values are compatible with polyamine levels of non-cancerous cells [17], it should be noted that several cancer cells display 10-fold higher intracellular polyamine concentrations [19]. Based on this rationale, the cytotoxic effect of SAMN@TA@BSAO was determined as a function of the enzyme nano-carrier concentration (5, 10, 25, 50, and 100 μg mL^−1^) on Caco-2 cells after 24 h incubation. Figure 4B shows that Caco-2 cell viability decreased as a function of SAMN@TA@BSAO concentration (red line), confirming the successful cell uptake of the nano-vehicle as well as its intracellular bioactivity. At low concentrations (less than 20 µg mL^−1^), the toxic effect of SAMN@TA@BSAO was limited and comparable to that of SAMN@TA. Cytotoxicity increased as a function of the nanohybrid concentration and, at SAMN@TA@BSAO concentrations higher than 50 µg mL^−1^, a saturation behavior was observed, likely due to the limited intracellular SPM concentration. Indeed, once the substrate was consumed at completion, any further uptake of immobilized enzyme produced no effect on cell viability. In Figure 4C, it is worth noting that when cell mortality was plotted against SAMN@TA@BSAO in a single-log plot, data were well interpreted by a sigmoidal dose–response curve (R^2^ = 0.99), typical of drug effect dynamics. The curve allowed the estimation of the half-maximal effective concentration (EC_50_) of the nanohybrid, resulting in 31 ± 1 µg mL^−1^, and reaching a cell viability of 53 ± 2% at high SAMN@TA@BSAO dosage. The time-dependent cell viability was examined at 12, 18, and 24 h using 35 µg mL^−1^ SAMN@TA@BSAO. Results shown in Figure 4D, red bars, indicate that treated cells’ viability decreased as a function of incubation time, showing negligible cytotoxicity at 12 h. SAMN@TA, under the same conditions, did not affect cell viability, as reported in Figure 4D, green bars.

### 2.4. Effects of SAMN@TA@BSAO Treatment on Oxidative Stress and Keap1/Nrf2 Pathway Activation in Caco-2 Cells

Intracellular reactive oxygen species (ROS) production was determined in Caco-2 cells in the absence and in the presence of 10 mM exogenous spermine, since it can enter the cells using specific transporters [44].

As reported in Figure 4E, in line with viability experimental results (Figure 4B), SAMN@TA@BSAO led to a significant increase in ROS production compared to controls. Notably, as expected, Figure 4F shows that the addition of 10 mM SPM to the culture medium led to a significant increase in ROS production.

When compared to the control (Figure 4G,H), samples treated with SAMN@TA@BSAO (Figure 4G,I) present clear signs of cell suffering, displaying translucent, granular, and denser nuclei (orange arrow) along with numerous vacuoles (green arrow).

**Figure 5:**
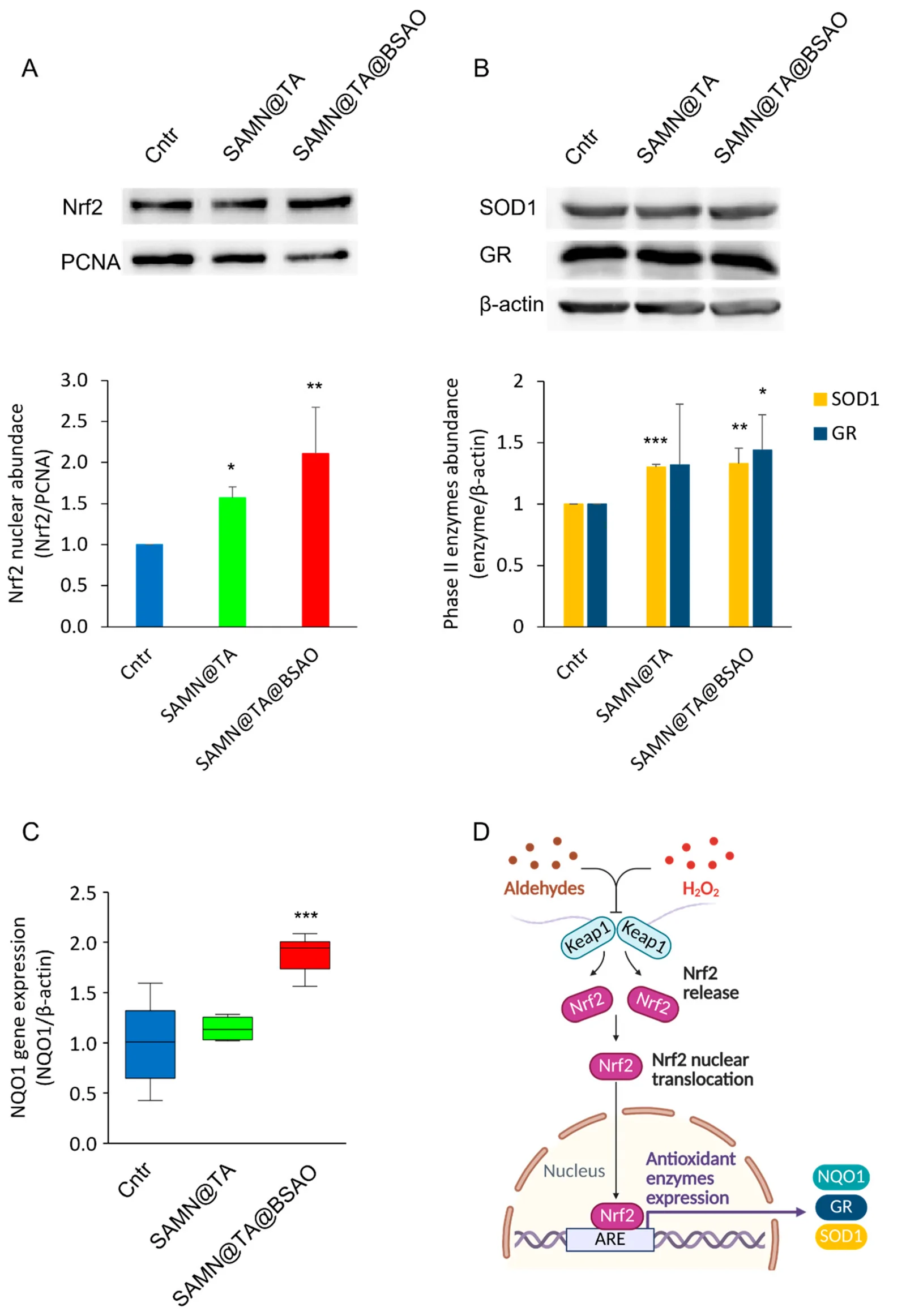
Study of the cellular pathway activated by the SAMN@TA@BSAO hybrid in Caco-2 cells. (**A**,**B**) Cells were treated with 35 µg mL^−1^ SAMN@TA or SAMN@TA@BSAO for 24 h. Nuclear fractions (**A**) and cell lysates (**B**) were obtained, and proteins were subjected to Western Blot (WB) detection. Densitometric analysis of nuclear factor erythroid-2-like factor 2 (Nrf2), glutathione reductase (GR), and superoxide dismutase 1 (SOD1) was performed, using proliferating cell nuclear antigen (PCNA) and β-actin as loading controls. (**C**) The gene expression of NADPH quinone oxidoreductase (NQO1) was evaluated in cDNA obtained from Caco-2 cells treated with SAMN@TA or SAMN@TA@BSAO nanohybrids (35 µg mL^−1^) for 24 h. β-actin was used as a reference. Data represent the mean of four independent experiments for each assay. Results were compared to the control. (*** *p* < 0.001, ** *p* < 0.01, * *p* < 0.05). (**D**) Schematic representation of the Keap1/Nrf2 cellular pathway activated by aldehydes and hydrogen peroxide. ROS act on Keap1, leading to the release of Nrf2, which migrates into the nucleus and activates the transcription of genes encoding the antioxidant enzymes GR, SOD1, and NQO1. Created in BioRender. BORTOLUZZI, M. (2026) https://BioRender.com/ht4h1tw.

Caco-2 cell response to SAMN@TA@BSAO uptake and the consequent oxidative stress in the presence of endogenous polyamines was studied by assessing the main signaling pathway involved in redox homeostasis regulation, namely, nuclear factor erythroid 2-related factor 2 (Nrf2) and Kelch-like ECH-associated protein 1 (Keap1), which constitute the main system modulating the antioxidant response (Figure 5D) [45,46]. As shown in Figure 5A, cells treated with SAMN@TA@BSAO showed a higher level of Nrf2 in the nucleus than untreated controls, suggesting that the Keap1/Nrf2 pathway was stimulated by the increased ROS production generated by nano-immobilized BSAO. Moreover, as shown by the experimental data reported in Figure 5B,C, the levels of the antioxidant enzymes, namely superoxide dismutase 1 (SOD1), glutathione reductase (GR), and NADPH quinone oxidoreductase (NQO1), were increased in cells treated with the SAMN@TA@BSAO nano-vehicle compared with untreated controls. The activation of the antioxidant signaling pathway explains the nearly constant decrease in cell viability observed at SAMN@TA@BSAO concentrations higher than 50 µg mL^−1^.

## 3. Discussion

When introduced in a biological system, the nanomaterials’ fate is predominantly governed by their hard corona, influencing their transport, accumulation, and toxicity [47]. Indeed, upon exposure to a physiological environment, a nanomaterial is enveloped in a dynamic layer of proteins, which evolves from a cloud of loosely bound proteins (soft corona) to a stable coating of selected macromolecules, strongly interacting with the inorganic surface (hard corona). This hard corona represents the ultimate interface between the nanomaterial and cells. Aiming at attaining higher nanomaterial biocompatibility and favorable cell recognition, a breakthrough approach consists of engineering nanoparticle surfaces with a rationally designed, functional hard protein corona [48]. Herein, the main novelty relies on the development of a tailored hard corona combining both stealth and biocatalytic features.

The coupling of BSAO and SAMN@TA takes place spontaneously through a mild wet reaction, and no significant structural adaptation is needed by BSAO to maximize the chemical contact with SAMN@TA. Indeed, the high-affinity self-assembly confers robustness to the nano-immobilized enzyme without compromising its biological activity. Additionally, the process endows SAMN@TA@BSAO with good overall colloidal stability, which has pivotal importance for real applicability in aqueous biological environments.

As reported in Section 2, despite being constituted of a bulky enzyme (BSAO MW 180 kDa), the engineered hard corona promotes the effective transport of SAMN@TA@BSAO into Caco-2 cells. Several possible cellular routes and uptake pathways have been proposed for nanoparticles, considering nanoparticle size, physicochemical properties, such as shape, electric charge, surface chemical structure, and, in particular, protein corona [49,50]. In this view, when compared to the uptake behavior of controls, that is, the unmodified SAMN@TA nano-carrier, SAMN@TA@BSAO was significantly dissimilar, showing an initially slower but continuous flow of the BSAO-based nano-vehicle into cells, well-described by a linear incorporation trend. Otherwise, SAMN@TA internalization exhibits saturation behavior, likely due to the occurrence of a corona composed of proteins coming from the cell culture medium. It is well-known that tannic acid has a proclivity to interact with proteins [39], likely explaining a different cellular uptake pathway [38,40].

The detection of internalized nano-vehicle was evidenced by confocal microscopy, exploiting the intrinsic luminescence of the inorganic core, SAMNs [30], enabling the tracking of the biomolecular cargo within cells. Acquired images demonstrated that SAMN@TA@BSAO crossed the cell membrane, forming vesicles. When compared to SAMN@TA, the enzyme-bearing nano-vehicle showed a higher ability to cross the simulated epithelium. In fact, experiments with endocytosis inhibitors showed that, differently from SAMN@TA, SAMN@TA@BSAO transport into cells was significantly more inhibited by CPZ, and to a lesser extent by CYP, prompting that cell uptake of the nano-immobilized enzyme was mainly due to clathrin-mediated endocytosis (CME). This suggests a specific recognition behavior by the cell membrane and plausibly explains the proclivity of the large hybrid SAMN@TA@BSAO to cross the biological barrier model (Figure 3). Indeed, CME was recently proposed as a key mechanism contributing to the transport of orally administered submicron material crossing the intestinal barrier [51]. Furthermore, the effect of the nano-immobilized BSAO on cells was investigated. Specifically, cell viability upon incubation in the presence of SAMN@TA@BSAO was modulated by nano-vehicle concentration, by treatment time, and, most importantly, by metabolic alterations, ranging from being safe at low polyamine levels to being systematically harmful for cells at high levels of polyamines. In fact, Caco-2 viability inversely depends on polyamine availability as substantiated by the saturation behavior observed in Figure 4B, which can likely be interpreted in terms of intracellular SPM depletion. Although the maximum effect of SAMN@TA@BSAO resulted in ca. 50% cell death, highlighting the remarkable cytotoxicity of the internalized nanohybrid, the overall trend suggests that it can be well tolerated in cells with normal polyamine levels, depending on SAMN@TA@BSAO dosage and treatment time.

**Figure 6:**
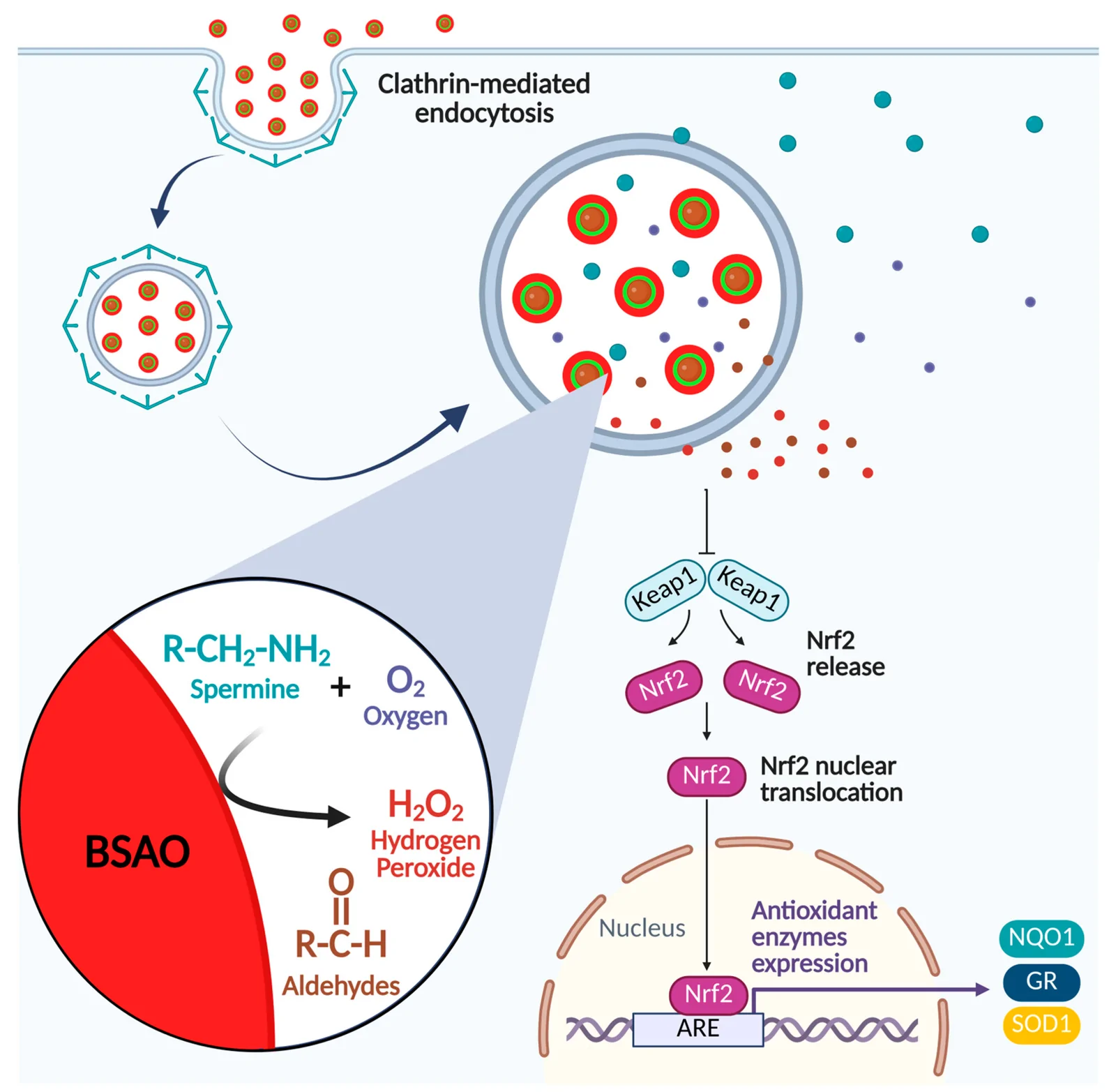
A schematic representation of the cellular pathway activated by the SAMN@TA@BSAO in Caco-2 cells. The hybrid is internalized by Caco-2 cells through clathrin-mediated endocytosis. Spermine molecules enter the vesicles and are oxidized by BSAO, leading to the generation of hydrogen peroxide and aldehydes. The resulting increase in reactive oxygen species (ROS) activates the Keap1–Nrf2 pathway, promoting the translocation of Nrf2 to the nucleus, where it drives the transcription of antioxidant genes, including GR, SOD1, and NQO1. Created in BioRender. BORTOLUZZI, M. (2026) https://BioRender.com/ht4h1tw.

The mechanism underlying SAMN@TA@BSAO nano-vehicle-induced cytotoxicity was also investigated (Figure 4E,F), and intracellular ROS generation was evaluated as a function of polyamine abundance, in particular spermine (SPM). Indeed, in the presence of SPM (10 mM), an increase in ROS production was observed, suggesting enhanced SAMN@TA@BSAO activity. The effect can be explained, at least partially, by the polyamine concentration-dependent generation of hydrogen peroxide (H_2_O_2_) during the enzymatic oxidation of polyamines [52,53]. Indeed, H_2_O_2_ and other ROS are highly reactive compounds that can interact with a wide range of cellular components, including nucleic acids, lipids, and proteins, thus impairing their structure and function. These reactive species can diffuse within the intracellular environment, reaching mitochondria and leading to oxidative damage [20,54]. These oxidative alterations can disrupt cellular homeostasis, damage membrane integrity, interfere with enzyme activity, and ultimately lead to cytotoxic effects, including cell dysfunction and death. Based on this observation, further investigation was dedicated to identify whether signaling pathways involved in redox homeostasis were activated. In this view, the saturation behavior observed in Figure 4B, besides being plausibly ascribable to the depletion of endogenous BSAO substrates, can be very likely linked to a cell adaptive response. This would result in the expression of protective proteins able to counteract the increase in oxidative stress induced by ROS generated through BSAO activity. Specifically, the main signaling pathway involved in the regulation of redox homeostasis was explored, namely the Keap1/Nrf2 pathway, which is modulated by nuclear factor erythroid 2-related factor 2 (Nrf2) and Kelch-like ECH-associated protein 1 (Keap1) [45,46]. Normally, Nrf2 is sequestered by Keap1 in the cytosol and degraded by the proteasome system. When Keap1 is modified by oxidants or radical species, it releases Nrf2, which enters the nucleus and interacts with a specific promoter known as the antioxidant response element (ARE). Nrf2-ARE complex modulates the expression of Phase II and antioxidant enzymes, such as superoxide dismutase-1 (SOD1), glutathione reductase (GR), and NADPH quinone oxidoreductase 1 (NQO1) [45,55]. Notably, when compared to controls, SAMN@TA@BSAO-treated cells showed a significantly greater accumulation of Nrf2 in the nucleus, indicating the activation of the Keap1/Nrf2 signaling pathway in response to the enhanced ROS generation promoted by nano-immobilized BSAO (Figure 5A). In line with this observation, the levels of Phase II and antioxidant enzymes, SOD1, GR, and NQO1, were also increased in cells treated with the SAMN@TA@BSAO nano-vehicle with respect to controls (Figure 5B,C). Overall, these results suggest that SAMN@TA@BSAO triggers oxidative stress while simultaneously activating cytoprotective pathways involved in redox homeostasis (Figure 6). Importantly, the sustained oxidative bioactivity observed in our system indicates that the nano-delivery and cellular absorption did not compromise BSAO structural integrity or catalytic site accessibility upon interaction with the biological milieu.

In conclusion, depending on intracellular polyamine content, SAMN@TA@BSAO appears to be an effective tool for inducing cell death. Notably, these substances are known to accumulate at high concentrations in several colorectal cancers [56]. At the same time, its biological activity can be further modulated by adjusting treatment time and nano-vehicle concentration.

Collectively, these results support SAMN@TA@BSAO as a proof of concept for a novel trimodal therapeutic agent, prompting its potential biomedical application and, therefore, further in vivo investigation.

## 4. Materials and Methods

All reagents were purchased at the highest commercially available purity and were used without further purification processes. SAMNs and the core–shell hybrid nanomaterial (SAMN@TA), constituted of SAMN (core) and tannic acid (TA, shell), were prepared according to Rilievo et al., 2022 [23]. BSAO was purified as already reported [23,57] and stored at −80 °C until use. Dulbecco’s modified Eagle’s medium (DMEM), fetal bovine serum (FBS), and penicillin-streptomycin were obtained from Gibco (Thermo Fisher Scientific, Waltham, MA, USA). All chemical reagents were purchased from Merck (Merck KGaA, Darmstadt, Germany).

### 4.1. SAMN@TA@BSAO Synthesis and Characterization

The surface concentration of BSAO was estimated by measuring the protein disappearance upon incubation with nanoparticles, considering the fraction of loosely bound macromolecules in the washing supernatants. Alternatively, the amount of nano-immobilized BSAO was determined by releasing the amine oxidase from SAMNs using sodium dodecyl sulfate. In all cases, protein concentration was assessed using the bicinchoninic acid (BCA) assay, using the Pierce™ BCA protein assay kit (Thermo Fisher Scientific), and all measurements were carried out in triplicate. The surface concentration of BSAO was 100 ± 5 mg g^−1^. The activity of bovine serum amine oxidases (BSAO) bound to the hybrid nanomaterial (SAMN@TA) was evaluated in 20 mM HEPES buffer, pH 7, containing 3 mM N,N-dimethylaniline, 4 mM 4-amino-antipyrine, and horseradish peroxidase (HRP) type II (50 U mL^−1^), as described by Rilievo et al., 2022 [23]. The enzyme activity was determined by continuously monitoring the H_2_O_2_ produced during spermine oxidation by the change in absorbance at 555 nm (using an extinction coefficient equal to 1.25 × 10^4^ M^−1^ cm^−1^). All measurements were carried out at room temperature (25 ± 1 °C). Circular dichroism (CD) analysis of BSAO, SAMN@TA, and SAMN@TA@BSAO was performed using a N_2_-flushed JASCO J-810 spectropolarimeter using a 2 mm quartz cuvette (Hellma Analytics, Müllheim, Germany). Measurements were performed in aqueous solutions containing 10 mM potassium phosphate buffer at pH 7. Transmission Electron Microscopy (TEM) micrographs were acquired using a Jeol JEM-2010 microscope (Jeol Ltd., Tokyo, Japan) operating at 200 kV with a point-to-point resolution of 1.9 Å. Prior to measurements, samples were dispersed in ethanol, and the suspension was treated using ultrasound for 10 min. A drop of diluted suspension was placed on a carbon-coated copper grid and allowed to dry via evaporation at room temperature.

The hydrodynamic size distribution and zeta potential were measured by Dynamic Light Scattering (DLS) using a ZEN3600 Zetasizer Nanoparticle analyzer coupled with a DTS1070 folded capillary cell at 25 ± 1 °C (Malvern Instrument, Malvern, UK).

The enzyme activity of the SAMN@TA@BSAO hybrid and parent BSAO, as a control, was performed in 10 mM Britton-Robinson buffer [58], pH 7.4, and constant ionic strength, using increasing concentrations of spermine as substrate. The enzyme activity was spectrophotometrically determined by measuring the H_2_O_2_ production rate, involving horseradish peroxidase (HRP) and a reduced dye [59]. These measurements were carried out at room temperature (22 ± 1 °C), monitoring the increase in the absorbance of the HRP-catalyzed secondary reaction product at 555 nm using a molar extinction coefficient of 1.25 × 10^4^ M^−1^ cm^−1^. Kinetic parameters (k_cat_ and K_M_) were calculated by fitting the Michaelis-Menten equation to the experimental data (rate of reaction vs spermine concentration) and resulted in 0.5 min^−1^ and 3.0 µM, corroborating that the macromolecules were well-preserved upon nano-immobilization [23]. The enzymatic activity of nano-immobilized BSAO was monitored for 2 months upon storage at 4 °C and remained well conserved, whereas the free enzyme tended to deteriorate. OriginLab software (version 7.5; OriginLab Corporation, Northampton, MA, USA) was used for the analysis of kinetic data. The kinetic constants were calculated by assuming an enzyme molecular weight of 180 kDa [60,61].

### 4.2. Evaluation of the Nanohybrid Effects on Cell Cultures

Caco-2 cells, derived from human colorectal adenocarcinoma, were kindly provided by the Department of Surgical Science, Oncology, and Gastroenterology (University of Padova, Italy). Cells were maintained in Dulbecco’s modified Eagle’s medium (DMEM), enriched with 10% fetal bovine serum (FBS), 10,000 units mL^−1^ of penicillin, and 1 mg mL^−1^ of streptomycin, at 37 °C in a humidified atmosphere containing 5% CO_2_. For this study, Caco-2 cells between the 35th and 60th passages were used.

#### 4.2.1. Evaluation of Polyamine Content in Caco-2 Cells

Cells (5 × 10^6^) were washed using 1X PBS, then centrifuged for 5 min at 500× *g*. The pellet was treated with 0.2 M perchloric acid, then tandem gas chromatography–mass spectrometry (GC-MS) analysis was performed assessing polyamine content in cells according to Di Fusco et al., 2011 [62].

#### 4.2.2. Cell Lysates

Caco-2 cells (4.5 × 10^6^ per well) were seeded in six-well plates and, after 48 h, treated with 35 µg mL^−1^ SAMN@TA or SAMN@TA@BSAO for Caco-2 cells. After 24 and 48 h, cells were collected, rinsed with 1 mL of 1X PBS and then lysed for 45 min at 4 °C with 100 µL modified RIPA buffer (150 mM NaCl, 1% Triton X 100, 0.1% SDS, 0.5% DOC, 1 mM NaF, 1 mM EDTA, 5 mM Tris/HCl (pH 7.4), 0.1 mM phenylmethanesulfonyl fluoride, PMSF) in the presence of protease inhibitor cocktail (Complete, Roche^®^, Basel, Switzerland) [63]. At the end of the incubation, cells were centrifuged at 11,600× *g* for 5 min at RT to discard the debris. Protein content was measured by applying the Lowry method [64].

#### 4.2.3. Estimation of SAMN@TA@BSAO Cellular Absorption

Determination of Iron Content in Caco-2 Cells Treated with SAMN@TA@BSAO

The uptake of SAMN@TA@BSAO and SAMN@TA nanohybrids by Caco-2 cells was measured by determining cell iron content, using an Inductively Coupled Plasma Optical-Emission Spectrometry (ICP-OES, Arcos by Spectro, Berwyn, IL, USA). Briefly, Caco-2 cells (4.5 × 10^6^ per well) were seeded in 6-well plates and treated with 35 µg mL^−1^ SAMN@TA and SAMN@TA@BSAO. After 24 h of treatment, cells were lysed as described in Section 4.2.2 and stored at −20 °C until being processed for ICP-OES analysis. Total iron content in each sample was determined after mineralization of the biological material with nitric acid.

Transepithelial Transport of SAMN@TA@BSAO Nanohybrid Through Caco-2 Cell Monolayers

The transepithelial transport of the SAMN@TA@BSAO nanohybrid was tested according to the protocol described by Tonolo et al. 2020, with some modifications [65]. Caco-2 cells (8 × 10^4^) were seeded on Transwell^®^ insert supports (0.4 µm pore sizes, 12 mm Ø, 1.12 cm^2^ growth surface, Corning Life Sciences, Tewksbury, MA, USA). To assess the formation and the integrity of the cell monolayer, transepithelial electrical resistance (TEER) was measured with a Millicell^®^ ERS2 volt-ohmmeter (EDM Millipore, Darmstadt, Germany). After 21 days of culture, only differentiated Caco-2 cell monolayers with transepithelial electrical resistance (TEER) values exceeding 1100 Ω cm^2^ were selected for investigating the nanohybrid’s transepithelial transport. The monolayer was gently washed three times with fresh DMEM containing 10% FBS. Then, the apical chamber medium was replaced with 0.5 mL of 35 µg mL^−1^ SAMN@TA or SAMN@TA@BSAO resuspended in DMEM, while 1.5 mL fresh medium was added to the basolateral chamber. Cells were incubated at 37 °C for 24 h. After incubation, the three Transwell compartments were collected separately. The medium from the apical chamber was transferred into a microcentrifuge tube, the membrane carrying the cell monolayer was removed and collected in a separate tube as the cellular compartment, and the medium from the basolateral chamber was transferred into a third tube. All samples were stored at −20 °C until ICP-OES analysis for iron quantification. The same analysis was also carried out using 10 µg mL^−1^ chlorpromazine (CPZ) and 5 µg mL^−1^ cytochalasin D (CYP), used as inhibitors of clathrin-mediated endocytosis and micropinocytosis/phagocytosis, respectively.

Confocal Microscopy Analysis of the Nanohybrids Localization

Caco-2 cells (4 × 10^4^) were seeded on cover glass (13 mm Ø, VWR, Radnor, PA, USA) and placed in 24-well plates (Sarstedt, Nümbrecht, Germany). After 48 h, cells were treated with 35 µg mL^−1^ SAMN@TA or SAMN@TA@BSAO for 24 h. Cells were fixed and stained for confocal microscopy imaging as follows. First, the medium was discarded, and cells were washed twice with 500 µL 1X PBS/well. Then, 300 µL well^−1^ of 2% paraformaldehyde (Sigma-Aldrich, St. Louis, MO, USA) was added. Cells were incubated at 4 °C for 30 min. Paraformaldehyde was removed, and cells were washed three times with 500 µL 1X PBS/well for 10 min at room temperature by gentle shaking. Then, fixed cells were incubated with 1:1000 Hoechst (Merck KGaA, Darmstadt, Germany) and 1 µM CellTracker Red CMTPX (Invitrogen, Waltham, MA, USA) at 37 °C for 30 min at RT in the dark. After three washes were carried out as already described (*vide supra*), the microscope coverslip slides (VWR International, Radnor, PA, USA) were treated with Mowiol (Calbiochem, San Diego, CA, USA) according to the manufacturer’s instructions and left to dry in the dark before imaging. Images were acquired by a Leica TCS SP5 confocal microscope equipped with a Leica HCX PL APO 40×/1.25–0.75 Oil CS objective. SAMNs possess an intrinsic luminescence (excitation at 488 nm, emission at 525 nm) [30], while Hoechst (Ex. 352 nm, Em. 454 nm) and CellTracker (Ex. 555 nm, Em. 602 nm) were used to mark the nuclei and cytoplasm, respectively.

### 4.3. Evaluation of SAMN@TA@BSAO Nanohybrid Effect on Caco-2 Cells

#### 4.3.1. Determination of Cell Viability in Caco-2 Cells in the Presence of SAMN@TA@BSAO Nanohybrid

The MTT assay for cellular metabolic activity, which is almost ubiquitous in studies of cell toxicity [66], was used to assess cell viability after exposure to SAMN-based nanohybrids. Caco-2 cells (10^4^ cells per well) were grown in a 96-well plate for 48 h [30], then 100 µL of 35 µg mL^−1^ SAMN@TA, 35 µg mL^−1^ SAMN@TA@BSAO, or complete medium for controls was added to Caco-2 cells and incubated for 12, 18, or 24 h. At the end of the incubation time, the medium was removed, and 100 µL MTT solution (0.5 mg mL^−1^) in 1X PBS was added to the cells. Then, the plate was placed in the dark at 37 °C and 5% CO_2_ for 3 h. The MTT solution was replaced by adding 100 µL of stop solution (90% isopropanol and 10% dimethyl sulfoxide). After 15 min at 37 °C, the absorbance (595–690 nm) was recorded using a Tecan plate reader (Tecan Infinite^®^ M200 PRO, Männedorf, Switzerland). Cell viability was reported as % of viable cells, where control cells were set as 100%.

#### 4.3.2. ROS Production Determination in Caco-2 Cells Treated with SAMN@TA@BSAO Nanohybrid

The production of intracellular ROS in Caco-2 cells exposed to SAMN@TA@BSAO was evaluated by using dihydrorhodamine 123, a fluorogenic probe (DHR123, Molecular Probes, Thermo Fisher Scientific, USA), according to Tonolo et al., 2024, with some modifications [67]. This molecule represents a chemically reduced form of R123 and freely diffuses into Caco-2 cells. Reactive oxygen species (ROS) oxidize DHR123, resulting in its conversion to the green fluorescent molecule R123 [68].

Cells (1 × 10^4^) were seeded in a 96-well plate and, after 48 h, treated with 35 µg mL^−1^ SAMN@TA or SAMN@TA@BSAO. After 24 h, cells were washed in HBSS 1X/10 mM glucose solution and loaded with 15 µM dye for 30 min in the dark at 37 °C. Afterward, cells were washed, and after adding 100 µL HBSS 1X/10 mM glucose solution to the wells, the reaction was evaluated following the increase in probe fluorescence (Ex. 485 nm, Em. 527 nm) with a plate reader (Tecan Infinite^®^ M200 PRO).

### 4.4. Determination of Antioxidant Signaling Pathway in Caco-2 Cells

#### 4.4.1. Western Blot Analysis of Proteins Involved in the Cell Antioxidant Response

The possible involvement of a Keap1/Nrf2 pathway regulating redox homeostasis was analyzed in Caco-2 cells by measuring the expression levels of type 1 superoxide dismutase (SOD-1), glutathione reductase (GR), and the nuclear transcription factor Nrf2. Caco-2 cells (4.5 × 10^5^ per well) were seeded in 6-well plates and, after 48 h, treated with 35 µg mL^−1^ SAMN@TA or SAMN@TA@BSAO. After 24 h, cells were lysed following the protocol reported in Section 4.2.2, and nuclear fractions were obtained according to Tonolo et al., 2024 [67]. Then, proteins were subjected to SDS-PAGE (10%) and subsequently to Western Blot (WB) analysis to define the expression level of SOD-1, GR, and Nrf2 in the nucleus. Nrf2 (A-10, sc-365949), SOD-1 (G-11, sc-17767), and GR (C-10, sc-133245) were purchased from Santa Cruz Biotechnology (Dallas, TX, USA), and β-actin, used as a loading reference, was obtained from Sigma-Aldrich (St. Louis, MO, USA, AC-15, A3854-200 UL). The anti-mouse secondary antibody was purchased from Invitrogen (Thermo Fisher Scientific, Waltham, MA, USA) and diluted 1:8000. Densitometric analysis of WB was carried out using Nine Alliance software (Mini 9 17.01 version, Uvitec Alliance, Cambridge, UK).

#### 4.4.2. Evaluation of NADPH Quinone Oxidoreductase 1 (NQO1) Gene Expression Levels

The levels of NQO1 gene expression, as a regulator of cell redox response, were evaluated with qRT-PCR, and β-actin was used as a reference. Caco-2 cells (5 × 10^5^) were seeded into six-well plates and, after 48 h, treated with 35 µg mL^−1^ SAMN@TA or SAMN@TA@BSAO. The mRNA was extracted using chloroform and precipitated with isopropanol according to Tonolo et al., 2022 [63]. The concentration of RNA was measured using a NanoDrop system (Thermo Fisher Scientific, Waltham, MA, USA), while 1 µg of RNA was subjected to reverse transcription using LunaScript™ RT SuperMix Kit (New England Biolabs, Ipswich, MA, USA). The target cDNA was amplified using Luna^®^ Universal qPCR Master Mix (New England Biolabs, Ipswich, MA, USA). Briefly, samples were subjected to an initial step of denaturation at 95 °C for 1 min, and then 42 cycles of amplification (15 s at 95 °C and 30 s at 60 °C) using the following primers: β-actin Fw: 5′-ACCTGACTGACTACCTCATGAAGA-3′, β-actin Rv: 5′-GCGACGTAGCACAGCTTCTC-3′, NQO1 Fw: 5′-GGA GAC AGC CTC TTA CTT GCC AAG-3′, NQO1 Rv: 5′-CCA GCC GTC AGC TAT TGT GGA-3′.

### 4.5. Statistical Analysis

Values were indicated as mean ± SD of at least three independent experiments. Statistical analyses were performed using the one-way ANOVA test with multiple comparison tests through the Tukey–Kramer method, and results with *p* < 0.05 were considered statistically significant. OriginPro software (OriginLab Corporation, Northampton, MA, USA) was used for the analysis.

## 5. Conclusions

The present study aimed at the fabrication of a catalytically active nano-drug for crossing the intestinal barrier and producing cytotoxic aldehydes and hydrogen peroxide by the oxidation of intracellular polyamines. For this purpose, thermodynamically driven by chemical-physical complementarity, BSAO was firmly immobilized onto SAMN@TA, a magnetic and luminescent carrier, leading to a stealth and bioactive hard protein corona. An intestinal model was employed to investigate the internalization of SAMN@TA@BSAO in Caco-2 cells, a human immortalized cell line derived from colorectal adenocarcinoma. Following cellular uptake, BSAO preserved its enzymatic activity, promoting oxidative stress and activating antioxidant defense pathways through the Keap1/Nrf2 signaling. Importantly, the resulting cytotoxic effect was long-lasting, dependent on both dose and exposure time, and, therefore, supporting the potential use of this nano-vehicle as a programmable therapeutic strategy to selectively target cells characterized by elevated polyamine levels. Notably, this study paves the way to in vivo tests and to the future employment of SAMN@TA@BSAO as a potential theragnostic tool.

## Data Availability

The original contributions presented in this study are included in the article. Further inquiries can be directed to the corresponding author.

## Supplementary Materials

The following supporting information can be downloaded at: https://www.mdpi.com/article/10.3390/ijms27167492/s1.

## Abbreviations

The following abbreviations are used in this manuscript:

ARE	Antioxidant response element
AOs	Amine oxidases
BSAO	Bovine serum amine oxidase
CD	Circular dichroism
CYP	Cytochalasin D
CPZ	Chlorpromazine
CME	Clathrin-mediated endocytosis
DLS	Dynamic light scattering
DMEM	Dulbecco’s modified Eagle’s medium
EC_50_	Half-maximal effective concentration
FBS	Fetal bovine serum
GC-MS	Gas chromatography—mass spectrometry
H_2_O_2_	Hydrogen peroxide
HRP	Horseradish peroxidase
ICP-OES	Inductively coupled plasma optical-emission spectrometry
Keap1	Kelch-like ECH-associated protein 1
MTT	3-(4,5-Dimethylthiazol-2-yl)-2,5-Diphenyltetrazolium Bromide
Nrf2	Nuclear factor erythroid 2-related factor 2
NQO1	NADPH quinone oxidoreductase
PA	Polyamine
PCNA	Proliferating cell nuclear antigen
PUT	Putrescine
ROS	Reactive oxygen species
SAMNs	Surface-active maghemite nanoparticles
SOD	Superoxide dismutase 1
SPD	Spermidine
SPIONs	Superparamagnetic iron oxide nanoparticles
SPM	Spermine
TA	Tannic acid
TEM	Transmission electron microscopy
TPQ	2,4,5-trihydroxyphenylalanine quinone
WB	Western Blot
