# Supplementary material for: Amino Oxidase Hard Protein Corona with Metabolic-Triggered Intracellular Biocatalysis
Source: Int J Mol Sci. 2026 Aug 21;27(16):7492. doi: 10.3390/ijms27167492 (PMC13513862; doi:10.3390/ijms27167492)
Supplement: Supplementary file 1 [file ijms-27-07492-s001.zip › ijms-4487205-supplementary.pdf]

Amino Oxidase Hard Protein Corona with Metabolic-Triggered Intracellular Biocatalysis

Federica Tonolo[1*], Mary Bortoluzzi[1], Graziano Rilievo[1], Alessandro Cecconello[2], Aura Cencini[1], Lavinia Rutigliano[3], Maria Pia Rigobello[4], Maria Luisa Di Paolo[5], Alberto Macone[6], Pasquale Fino[7], Enzo Agostinelli[7,8], Massimiliano Magro[1*], Fabio Vianello[1,8]

[1] Department of Comparative Biomedicine and Food Science, University of Padua, Viale dell'Università 16, 35020 Legnaro, Italy.
[2] Department of Molecular and Translational Medicine, University of Brescia, Viale Europa 11, 25123 Brescia, Italy.
[3] Department of Molecular Medicine, Sapienza University of Rome, Viale Regina Elena 291, 00161 Rome, Italy.
[4] Department of Biomedical Sciences, University of Padua, Via Ugo Bassi 58/b, Padova, Italy.
[5] Department of Molecular Medicine, University of Padua, Via Gabelli 63, 35121 Padova, Italy.
[6] Department of Biochemistry Science "A. Rossi Fanelli", La Sapienza University of Rome, Viale Regina Elena 332, 00161 Rome, Italy.
[7] Department of Sensory Organs, La Sapienza, University of Rome, Policlinico Umberto I, Viale del Policlinico 155, 00161 Rome, Italy.
[8] International Polyamines Foundation 'ETS-ONLUS', Via del Forte Tiburtino 98, 00159 Rome, Italy.

*Correspondence: federica.tonolo@unipd.it (F.T.); massimiliano.magro@unipd.it (M.M.)

Supplementary Information

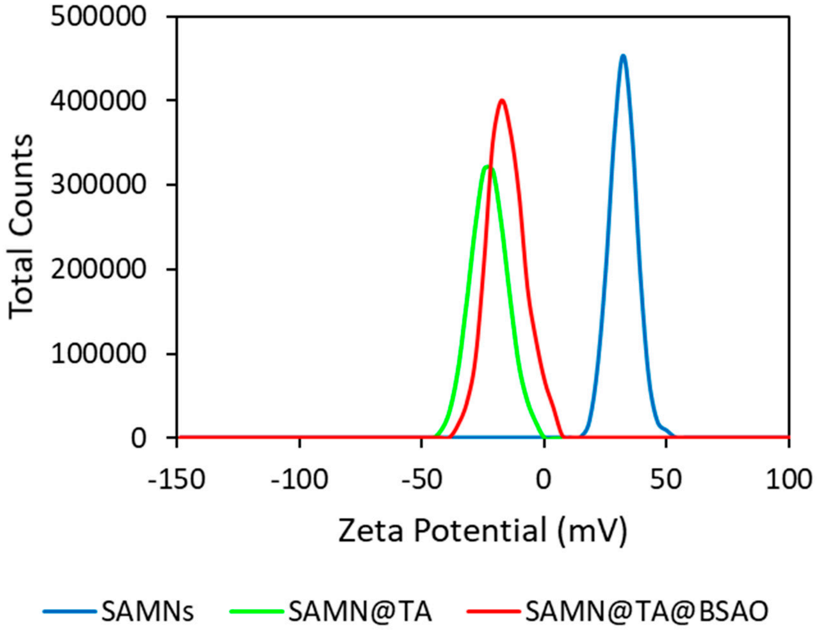

Figure S1: Comparison of the zeta potential of SAMNs, SAMN@TA and SAMN@TA@BSAO.
